# Neuron-Astrocyte Interactions in Parkinson’s Disease

**DOI:** 10.3390/cells9122623

**Published:** 2020-12-07

**Authors:** Ikuko Miyazaki, Masato Asanuma

**Affiliations:** Department of Medical Neurobiology, Graduate School of Medicine, Dentistry and Pharmaceutical Sciences, Okayama University, Okayama 700-8558, Japan; asachan@cc.okayama-u.ac.jp

**Keywords:** astrocyte, Parkinson’s disease, dopaminergic neuron, neuroinflammation, neuroprotection, α-synuclein, mitochondria

## Abstract

Parkinson’s disease (PD) is the second most common neurodegenerative disease. PD patients exhibit motor symptoms such as akinesia/bradykinesia, tremor, rigidity, and postural instability due to a loss of nigrostriatal dopaminergic neurons. Although the pathogenesis in sporadic PD remains unknown, there is a consensus on the involvement of non-neuronal cells in the progression of PD pathology. Astrocytes are the most numerous glial cells in the central nervous system. Normally, astrocytes protect neurons by releasing neurotrophic factors, producing antioxidants, and disposing of neuronal waste products. However, in pathological situations, astrocytes are known to produce inflammatory cytokines. In addition, various studies have reported that astrocyte dysfunction also leads to neurodegeneration in PD. In this article, we summarize the interaction of astrocytes and dopaminergic neurons, review the pathogenic role of astrocytes in PD, and discuss therapeutic strategies for the prevention of dopaminergic neurodegeneration. This review highlights neuron-astrocyte interaction as a target for the development of disease-modifying drugs for PD in the future.

## 1. Introduction

Parkinson’s disease (PD) is a progressive neurodegenerative disease mainly characterized by the loss of dopaminergic neurons in the substantia nigra pars compacta (SNpc). Consequently, dopamine (DA) deficiency in the striatum causes motor symptoms, such as akinesia/bradykinesia, tremor, rigidity, and postural instability. PD patients also exhibit non-motor symptoms, such as hyposmia, autonomic disturbance, depression, and REM sleep behavior disorder (RBD), which precedes motor symptoms [1]. Pathologically, neurodegeneration accompanied by an accumulation of α-synuclein, Lewy bodies and neurites, is observed in the central and peripheral nervous systems of sporadic PD patients [2,3]. Currently, it is hypothesized that PD pathology propagates from the enteric nervous system (ENS) to the central nervous system (CNS) via the vagal nerve [4,5]. Although the mechanism of neurodegeneration in PD remains unknown, various pathogenic factors, such as oxidative stress, neuroinflammation, α-synuclein toxicity and mitochondrial impairment, and neuron vulnerability, are thought to drive apoptosis. In addition, there is a consensus that non-neuronal cells contribute to the progression of PD pathology [6,7,8].

Astrocytes are the most numerous glial cells in the CNS and surrounding neurons. Astrocytes have been shown to protect neurons by the release of neurotrophic factors, the production of antioxidants, and the disposal of neuronal waste products, including aggregated α-synuclein and damaged mitochondria [9,10]. In the brains of patients with neurodegenerative diseases, astrocytes are known to migrate and become hypertrophic, that is, reactive astrocytes. The role of these reactive astrocytes is controversial; it is not clear whether these cells are neuroprotective or neurotoxic [11]. Recently, the classification of reactive astrocytes has been proposed as harmful A1 and protective A2 astrocytes [12]. It is reported that A1 astrocytes lose their characteristic functions, including neuroprotective and supportive property, and contribute to neuronal death in PD [12]. On the other hand, we previously demonstrated that reactive astrocytes produce antioxidative molecules in response to oxidative stress and protect dopaminergic neurons in PD models [13]; however, we did not characterize the types of reactive astrocytes. In this review, we summarize the interaction of astrocytes and dopaminergic neurons, and discuss the pathogenic role of astrocytes in PD. We also review the therapeutic strategies used to prevent dopaminergic neurodegeneration by upregulating astrocytic neuroprotective properties. Understanding the multiple functions of astrocytes and the interaction of astrocytes and neurons in neurological conditions will aid in the development of a disease-modifying drug for PD in future studies.

## 2. Interactions of Astrocytes and Dopaminergic Neurons

### 2.1. Dopaminergic Transmission

Astrocytes are located in synaptic regions; the perisynaptic processes of the cells occur in both presynaptic terminals and postsynaptic structures, whose construction is known as the “tripartite synapse” [14,15]. The neurotransmitters released from the presynaptic terminals interact with specific receptors located in both the postsynaptic neurons and astrocytes. Astrocytes express various receptors and transporters. Our previous studies demonstrated the expression of DA receptors, DA transporter (DAT), L-type amino acid transporter 1, and its covalently linked ancillary subunit 4F2hc in the basal ganglia [16,17]. Dopaminergic neurons extend their axons to the striatum and release DA in the striatum; indeed, a large quantity of DA is contained in the brain. In our studies, striatal astrocytes were found to express DA D1-, D3-, D4-, and D5-receptors and D4-mediated signal transduction in response to DA [17]. In addition, DA and L-dopa were taken up into striatal astrocytes in parkinsonian model rats after L-dopa treatment. While striatal astrocytes metabolize DA to DOPAC, the cells held L-dopa intracellularly and released L-dopa in response to a reduction in its concentration in the extracellular space [16]. These results suggest that striatal astrocytes could act as a reservoir for L-dopa in parkinsonism. Furthermore, recent studies demonstrated that adenosine A2A and DA D2 receptors were co-expressed on the processes of striatal astrocytes, and that A2A-D2 receptor-receptor interaction controlled glutamate release from astrocytes [18]. The receptor-receptor interaction is based on heteromerization of A2A and D2 receptors at the plasma membrane of striatal astrocytes [19]. The A2A-D2 heteroreceptor complexes in the striatum are important subject and lead to a new perspective on molecular mechanisms underlying PD pathophysiology and drug-induced dyskinesia. Therefore, the new astrocyte-related aspects of glutamatergic regulation in intercellular communication between neurons and astrocytes via A2A-D2 heteromer can contribute to the development of new pharmacological strategies for PD treatment.

### 2.2. Release of Neurotrophic Factors

Astrocytes secrete neurotrophic factors, such as glial cell line-derived neurotrophic factor (GDNF), brain-derived neurotrophic factor (BDNF), nerve growth factor (NGF), basic fibroblast growth factor (bFGF, FGF-2), cerebral dopamine neurotrophic factor (CDNF) and its homolog mesencephalic astrocyte-derived neurotrophic factor (MANF). These paracrine molecules bind to their target receptors in dopaminergic neurons to ensure correct neuronal development, differentiation and survival.

GDNF is a member of the transforming growth factor (TGF)-β superfamily, which promotes the survival and morphological differentiation of mesencephalic dopaminergic neurons [20,21]. Therefore, GDNF has been focused on as a useful molecule for PD therapy [22]. GDNF is reported to be mainly expressed in neurons, but not in astrocytes, under normal conditions; conversely, GDNF is upregulated in astrocytes in diseased brains [23]. Nakajima et al. [24] reported that astrocytes received signals from damaged neurons and produced GDNF to protect neurons against oxidative stress. In addition, it is reported that DA receptor activation, especially D1 receptor, increases GDNF expression in astrocytes [25,26].

Basic FGF is predominantly synthesized and secreted by astrocytes in the adult brain and promotes the growth and survival of neurons [27,28,29,30]. Indeed, mesencephalic neurons show enhanced growth and survival when cultured with astrocytes. Engele and Bohn [31] reported that mesencephalic astrocytes provide neurotrophic bFGF for central dopaminergic neurons. It has also been reported that the survival and differentiation of mesencephalic DA neurons are promoted by DA-mediated FGF-2 induction in striatal astrocytes [32]. The stimulation of DA D1 or D2 receptors induces FGF-2 biosynthesis and secretion in astrocytes [33,34]. Proia et al. [35] demonstrated that astrocytes shed bFGF-containing extracellular vesicles to extracellular spaces. It is also reported that bFGF induces transcriptional activation of GDNF in astrocytes [36].

CDNF and MANF were initially discovered as secreted proteins with trophic activity. MANF was first identified in the culture medium of a rat type-1 astrocyte ventral mesencephalic cell line, which provide protection for embryonic mesencephalic DA neurons in vitro [37]. CDNF is a vertebrate-specific paralogue of MANF. CDNF and MANF exert neuroprotective effects, especially in lesioned DA neurons. A single intrastriatal injection of CDNF or MANF was found to improve locomotor behavior and rescue dopaminergic neurons in the SNpc of 6-hydroxydopamine (6-OHDA)-induced PD models [38,39]. These neuroprotective effects of CDNF were also observed in 1-methyl-4-phenyl-1,2,3,6-tetrahydropyridine (MPTP)-injected animals [40]. Thus, MANF and CDNF are potent factors, providing protection and enhancing the repair of DA neurons. Although the mechanisms underlying the neuroprotective effects of CDNF and MANF are not well understood, these proteins are thought to be involved in the regulation of endoplasmic reticulum (ER) function and homeostasis [41,42]. Both proteins uniquely contain an N-terminal signal peptide that directs them to the ER and a C-terminal KDEL-like ER-retention signal. Both CDNF and MANF localize in the ER of healthy cells, and when the N-terminal signal peptide is cleaved, these proteins can be secreted [43]. Interestingly, CDNF and MANF function to protect cells against ER stress when they are expressed inside cells, whereas classical neurotrophic factors, including GDNF, are usually inactive inside cells. In the case of protein misfolding in the ER, the unfolded protein response (UPR) is activated. Both CDNF and MANF act as modulators of UPR. Furthermore, recent studies have demonstrated the anti-inflammatory role of CDNF and MANF; these proteins antagonize the secretion of pro-inflammatory factors such as interleukin (IL)-1β and tumor necrosis factor (TNF)-α [42].

### 2.3. Production of Antioxidative Molecules

Glutathione (GSH) is a well-known intrinsic antioxidant against reactive oxygen species (ROS). Cysteine is the rate-limiting precursor for GSH synthesis [44]. However, extracellular cysteine is readily auto-oxidized to cystine, as neurons lack a cystine transport system [45]. Astrocytes take up cystine via cystine/glutamate exchange transporter (xCT), reduce it to cysteine to synthesize GSH, and release it via the transporter multidrug resistance protein 1 (MRP1) [46,47]. The extracellularly released GSH is used to reduce cystine to cysteine via thiol/disulfide exchange reactions. This is followed by the uptake of cysteine by neighboring neurons for GSH synthesis [47,48,49]. In another way, astrocyte-released GSH is degraded by a peptidase, resulting in the production of the dipeptide cysteinylglycine (CysGly). It is reported that neurons also take up CysGly for GSH synthesis [44,50]. Therefore, GSH synthesis in neurons is dependent on the expression of xCT and GSH synthesis in astrocytes. GSH is a very important molecule especially for dopaminergic neurons, which contain DA as a neurotransmitter. Although DA stored inside synaptic vesicles is stable, cytosolic DA outside of vesicles is easily metabolized via monoamine oxidase (MAO) or by auto-oxidation to produce cytotoxic ROS [51]. Moreover, reactive quinones, such as DA quinones, are produced by the spontaneous oxidation of free cytosolic DA in dopaminergic neurons [52,53]. The generated DA quinones can covalently react with the sulfhydryl residues on important proteins for dopaminergic neurons, including tyrosine hydroxylase (TH), DAT and parkin to form quinoproteins, resulting in the dysfunction of these proteins [54,55,56,57]. Therefore, DA quinone is considered to exert DA neuron-specific oxidative stress [58]. GSH competes with the sulfhydryl group on cysteine in functional proteins to prevent the formation of quinoprotein [59]. Therefore, GSH acts as an antioxidant by quenching not only general ROS but also DA quinones [9]. Taken together, the cysteine supply from astrocytes to neurons plays a pivotal role in the survival of DA neurons.

Metallothioneins (MTs) are cysteine-rich proteins possessing antioxidative, anti-apoptotic, and anti-inflammatory properties [60]. MTs bind to metals such as zinc (Zn), copper (Cu), and cadmium via their cysteine-rich structure and function in metal homeostasis and detoxification [61]. Under oxidative stress, Zn is released from MTs and transferred to Zn-required transcription factors to regulate the expression of several genes involved in the antioxidant pathways [62]. In mammalian tissue, the MT family comprises four isoforms: MT-1, MT-2, MT-3, and MT-4. The two abundant isoforms, MT-1 and -2 (MT-1/-2), are expressed in most organs including the brain [60]. MT-1/-2 are produced primarily by astrocytes [13,63,64], and extracellular MTs secreted from astrocytes mediate neuronal survival and axonal regeneration [65,66]. The abundant thiol groups of MT-1/-2 allow them to exert radical-scavenging properties [60]. Our previous studies demonstrated that MT-1 quenched DA semiquinones in vitro [67], and that astrocyte-derived MT-1/-2 protected dopaminergic neurons against DA quinone toxicity both in vitro and in vivo [13]. Thus, MTs produced by astrocytes play a crucial role in the neuroprotection of DA neurons. It is known that secreted MTs bind to surface receptors belonging to the family of low-density lipoprotein receptor-related proteins (LRP), such as LRP-2/megalin and LRP-1, by which MTs can be taken up by neurons via endocytosis, activate the signal transduction pathways of neurite outgrowth, and exert protective actions [68].

Nuclear factor erythroid 2-related factor 2 (Nrf2) is a transcription factor that induces the expression of various antioxidant genes, such as GSH synthesizing enzyme glutamate-cysteine ligase, quinone reductase-1 (NQO-1), and MT-1/-2, by binding to the antioxidant response element (ARE) [13,69]. Nrf2 is bound in the cytoplasm by its repressor protein Kelch-like-ECH-associated-protein 1 (Keap1) and is constitutively degraded by proteasomes under normal conditions [70,71]. When exposed to electrophiles and ROS, the cysteine residues of Keap1 are oxidized, leading to conformational changes, which results in the release of Nrf2, followed by the nuclear translocation and activation of phase II antioxidant enzyme gene expression [72,73]. Phase II genes provide cellular defense by scavenging ROS, detoxifying electrophiles and xenobiotics, and maintaining intracellular antioxidative properties. Nrf2 is mainly expressed in astrocytes; Nrf2-regulated genes are preferentially activated in astrocytes, which consequently exhibit more efficient detoxification and antioxidant defenses than neurons [69]. Indeed, Nrf2-induced molecules, such as GSH-related enzymes and MTs, are higher in astrocytes than in neurons. A number of studies have demonstrated that the activation of Nrf2 in astrocytes plays a major role in protecting dopaminergic neurons from oxidative stress [13,69,74,75,76].

DJ-1 gene deletions and point mutations have been identified as one of the causes of early-onset autosomal recessive PD (AR-PD) (PARK7) [77]. DJ-1 is known to play a role in oxidative stress responses in cells, and is thought to be important in the preservation of neuronal viability [78]. DJ-1 is mainly expressed in astrocytes in the human brain, acting as a sensor of oxidative stress [79,80]. Reactive astrocytes enhance DJ-1 expression in response to oxidative stress and release the protein extracellularly to protect neurons [81]. Clements et al. [82] reported that Nrf2 protein without intact DJ-1 is unstable, with lower basal or inducible levels of transcriptional responses as a result. These observations suggest that DJ-1 stabilizes the antioxidant transcriptional regulation of Nrf2. Furthermore, DJ-1 has been identified as a critical molecule for mitochondrial function [83,84]. In the presence of an oxidative environment, DJ-1 plays an important role in maintaining mitochondrial function, as well as PTEN-induced kinase 1 (PINK1)-parkin pathway [84].

### 2.4. Rescue of Mitochondria in Dopaminergic Neurons

The direction of human pluripotent stem cells (iPSCs) into dopaminergic neurons is important for the identification of the underlying neuropathology, and thus for the selection of an adequate replacement therapy for PD. Recent studies have reported that astrocytes play an important role in the differentiation and maturation of dopaminergic neurons by rescuing mitochondria. Du et al. [85] reported that astrocytes restored mitochondrial function and dynamics during the differentiation of human iPSC-derived DA neurons. In addition, Cheng et al. [86] demonstrated that iPSC-derived astrocytes could act as mitochondrial donors to injured dopaminergic neurons, preventing neurodegeneration. Astrocytic mitochondria were found to be internalized by injured DA neurons via a phospho-p38 dependent pathway, indicating the significance of astrocytes for cellular therapy for PD.

### 2.5. Disposal of Neuronal Waste Products

The aggregated and insoluble fibrillar form of α-synuclein constitutes a major component of Lewy bodies and neurites in neurodegenerative diseases, such as PD, dementia with Lewy bodies, and multiple system atrophy [87,88]. Therefore, the accumulation of aggregated α-synuclein is associated with the pathogenesis of PD, and the efficient clearance of aggregated α-synuclein represents a potential approach in PD therapy. α-Synuclein is a cytosolic protein, but it can be released from neuronal cells into the surrounding extracellular space via exocytosis [89]. Several studies have reported that extracellular α-synuclein contains aggregated forms, and that misfolding and aggregation facilitate the release of α-synuclein from neuronal cells [90]. The uptake of extracellular α-synuclein occurs via endocytosis in neurons and glial cells [91]. In particular, microglia and astrocytes are able to clean extracellular α-synuclein aggregates via internalization and degradation [92,93,94]. Recently, astrocytes were reported to engulf and degrade α-synuclein aggregates via proteasome and autophagy pathways [95], suggesting that astrocytes exert dopaminergic neuroprotection by clearing excess extracellular toxic α-synuclein.

Damaged mitochondria are removed via mitophagy to maintain the quality of mitochondria [96,97,98]. This maintenance is important not only to ensure proper bioenergetic function, but also to prevent the release of ROS, which leads to cell death. In particular, mitophagy is essential for the health of DA neurons because mitochondrial impairment is a key feature of PD. Mitochondrial degradation is generally thought to be a cell-autonomous process. Recently, it was reported that damaged mitochondria are internalized and degraded by surrounding astrocytes [99]. Morales et al. [94] demonstrated that the damaged mitochondria of degenerating dopaminergic terminals were transferred to striatal astrocytes for their degradation. The authors also reported that striatal astrocytes engulf dopaminergic debris, which contains TH, DAT, and α-synuclein [100]. Taken together, the phagocytic activity of astrocytes is important for cleaning the dopaminergic debris produced by the degeneration of striatal dopaminergic nerve terminals.

## 3. Histology of Astrocyte in Parkinsonism

### 3.1. Astrocyte Reaction

Reactive astrocytes are characterized by the hypertrophy, proliferation, and up-regulation of the expression of cytoskeletal proteins, namely glial fibrillary acidic protein (GFAP). Generally, in pathological conditions, the accumulation of reactive astrocytes is detected in the brain, especially in injured areas. However, post-mortem cases of PD have been found to exhibit a mild increase in the number of astrocytes and the immunoreactivity for GFAP in the SNpc [101,102]. In addition, some reports have found that the number and morphology of astrocytes are not changed in the PD brain [103,104]. Damier et al. [105] reported that the density of GFAP-positive cells in the different dopaminergic areas in control brains was significantly correlated with the intensity of neuronal injury in PD brains. These results suggest that DA neurons within areas poorly populated with astrocytes tend to degenerate. In the brain of parkinsonian models, the accumulation of reactive astrocytes (i.e., astrogliosis) was dramatic; animals injected with 6-OHDA or MPTP have been found to exhibit a prominent increase in the number and reactivity of GFAP in the striatum and SN [106,107]. Although the role of reactive astrocytes is not well understood, several studies have found that neuroprotective molecules, such as antioxidants, are detected specifically in GFAP-positive astrocytes [13,64]. Taken together, the absence of obvious astrogliosis in PD brains is indicative of astrocyte dysfunction, leading to dopaminergic neurodegeneration.

### 3.2. Expression and Inclusion of α-Synuclein in Astrocytes

The neuropathological hallmark of sporadic PD is the formation of α-synuclein-immunoreactive inclusions. Various studies have reported that α-synuclein-containing inclusions were detected not only in neurons but also in astrocytes in post-mortem PD brains [108,109]. In addition, it has been reported that neuronal loss and the presence of cytoplasmic inclusions in neuronal cells are accompanied by presence of astrogliosis. Braak et al. [108] reported that large numbers of α-synuclein-positive astrocytes appeared in stages 4–6 of Braak’s PD staging and were concentrated in prosencephalic sites, including the amygdala, thalamus, septum, striatum, claustrum, and cerebral cortex. Furthermore, they demonstrated that the density of α-synuclein-positive astrocytes exerted an effect similar to that of the neuronal constituents of the cortex; the astrocytic reaction also accompanied the occurrence of Lewy bodies and neurites in the projection neurons of the thalamic intralaminar nuclei. Wakabayashi et al. [110] reported that α-synuclein-positive inclusions were present in the astrocytes and oligodendrocytes of the SN in patients with PD. Interestingly, the number of α-synuclein-positive inclusions within SNpc astrocytes is positively correlated with the severity of dopaminergic neuronal loss in the SNpc. As mentioned above, misfolded or aggregated α-synucleins are released from neurons, taken up by astrocytes, and degraded [90,92]. These observations suggest that the dysfunction of astrocytic clearance of α-synuclein aggregation promotes neurodegeneration in neurological conditions. Several studies have reported neuron-to-neuron or neuron-to-astrocyte spreading of the α-synuclein aggregates via a ‘‘prion-like’’ mechanism in cellular and animal models [111,112]. Moreover, di Domenico et al. [113] recently demonstrated that generated iPSC-derived astrocytes from familial PD patients with leucine-rich repeat kinase 2 (LRRK2) mutations induced the accumulation of α-synuclein aggregates in astrocytes, followed by the propagation of toxic aggregates to dopaminergic neurons. Thus, these neurons were abnormal and displayed morphological signs of neurodegeneration, suggesting that pathogenic crosstalk occurs between astrocytes and neurons.

## 4. Neurotoxic Role of Astrocyte in Parkinsonism

### 4.1. Neuroinflammation

**Figure 1:**
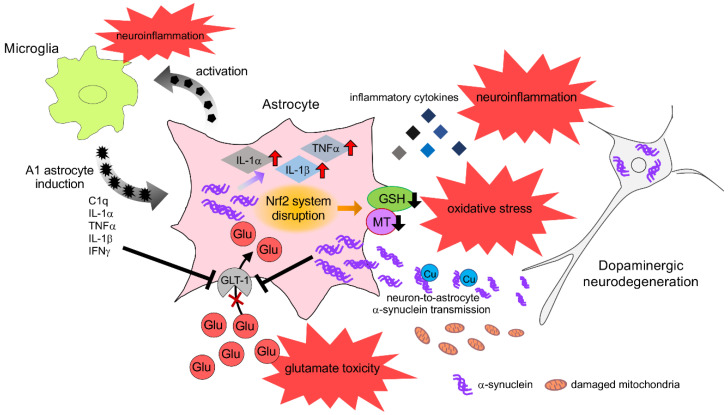
Neuron-astrocyte interaction under neuropathological conditions. Neurotoxic A1 astrocytes, induced by activated microglia, upregulate proinflammatory factors, such as IL-1α, IL-1β and TNF-α and produce neuroinflammation. Microglia-released proinflammatory mediators, such as TNF-α, IL-1β, and IFN-γ, inhibit glutamate uptake in astrocytes leading to glutamate toxicity. Nrf2 system disruption in astrocytes leads to decrease in antioxidative molecules, which results in oxidative stress. Damaged neurons release aggregated α-synuclein and damaged mitochondria. Neuron-to-astrocyte transmission of α-synuclein, followed by its accumulation and deposition in astrocytes, produces proinflammatory cytokines and impairs glutamate uptake via GLT-1.

Chronic neuroinflammation is consistently associated with the pathophysiology of PD. It is well known that activated glial cells release pro-inflammatory and neurotoxic factors that induce neuronal damage and neurodegeneration [114,115]. Indeed, microglial activation and increased levels of pro-inflammatory cytokines, such as TNF-α, IL-1β, and IL-6, were observed in the SN of PD patients post-mortem, as well as in animal models [116,117]. Various studies have revealed that inflammation promotes dopaminergic neurodegeneration; the intranigral injection of lipopolysaccharide, an endotoxin from Gram-negative bacteria, induces microglial activation and dopaminergic neuronal loss [118], while transgenic mice expressing TNF-α specifically in the brain induced dopaminergic neurotoxicity [119]. These studies suggest that DA neurons are more susceptible to inflammatory stimuli. Astrocytes also contribute to neuroinflammation. Recently, reactive astrocytes have been classified into two types, A1 and A2. A1 astrocytes highly upregulate many toxic genes, suggesting that A1 astrocytes may be harmful, while A2 astrocytes upregulate many neurotrophic factors, suggesting that A2 astrocytes are protective [12]. Furthermore, Liddelow et al. reported that neurotoxic A1 astrocytes were induced by activated microglia via secretion of cytokines IL-1α, TNF, and C1q. They also showed that A1 astrocytes upregulated proinflammatory factors, such as IL-1α, IL-1β, and TNF-α. In addition, A1 astrocytes were found to lose the ability to promote neuronal survival, outgrowth, synaptogenesis, and phagocytosis, and induced the death of neurons and oligodendrocytes. In addition, Joshi et al. [120] demonstrated that fragmented mitochondria released from microglia triggered an A1 astrocytic response and propagated inflammatory neurodegeneration. These findings suggest that microglia provoke astrocytes to induce an inflammatory response. Conversely, it has also been reported that astrocytes control microglial activation and microglia-induced neuroinflammation [121,122], suggesting that astrocyte-microglia intimate crosstalk mediates neuroinflammation (Figure 1).

The nucleotide-binding oligomerization domain leucine-rich repeat and pyrin domain-containing protein 3 (NLRP3) inflammasome has been reported to play a key role in neuroinflammation [123]. The activation of the NLRP3 inflammasome in microglia and astrocytes leads to the production of IL-1β and IL-18 pro-inflammatory cytokines [124,125]. Indeed, both levels of IL-1β and IL-18 are higher in the cerebrospinal fluid of PD patients than in healthy controls [126]. In addition, it has been reported that the inhibition of astrocytic NLRP3 inflammasome activation and subsequent IL-1β production exhibits neuroprotective effects [127,128]. Taken together, IL-1β produced by the astrocytic NLRP3 inflammasome is thought to be crucial in the pathogenesis of PD.

Glutamate is the main excitatory neurotransmitter in the CNS. Released glutamate in the synaptic cleft is taken up through Na^+^-dependent excitatory amino acid transporters EAAT1 (GLAST) and EAAT2 (GLT-1), which are predominantly expressed in astrocytes [129]. Therefore, astrocytes play an important role in preventing glutamate excitotoxicity. Various studies have reported that inflammation affects glutamate-removing activity in astrocytes [130,131,132]. Anti-inflammatory drugs influenced glutamate handling in the human astrocytoma cell line U-373MG, suggesting that inflammatory mediators could increase glutamate release or impair glutamate uptake [133]. In addition, proinflammatory mediators such as TNF-α, IL-1β, and IFN-γ released from microglia influence glutamatergic transmission. Zou et al. [132] reported that TNF-α potentiated glutamate toxicity by inhibiting glutamate uptake in astrocytes. In addition, Takaki et al. [134] demonstrated that glutamate derived from activated microglia downregulated the expression of astrocytic glutamate transporters as a result of the elevation of intracellular glutamate levels in astrocytes. These data indicate that TNF-α and glutamate can act synergistically to induce neuronal cell death in inflammatory conditions.

Other molecules in astrocytes can also contribute to neuroinflammation such as DA D3 receptor signaling and nuclear factor kappa B (NF-κB) signaling [135,136,137]. Montoya et al. [137] demonstrated that the genetic deficiency of DA D3 receptor signaling changed the astrocyte phenotype to unresponsive to LPS-induced neuroinflammation. In addition, it has been reported that NF-κB signaling in astrocytes mediates neuroinflammation, followed by neuronal loss, in MPTP-injected PD models [135,136].

### 4.2. α-Synuclein Toxicity

The neuron-to-astrocyte transmission of α-synuclein, followed by its accumulation and deposition in astrocytes, can cause neuroinflammation and contribute to neurodegeneration in PD. It has been reported that astrocytes with accumulated α-synuclein produce proinflammatory cytokines, including IL-1, IL-6, and TNF-α, as well as chemokines, such as CXCL1 and CX3CL1 [112]. This α-synuclein-induced proinflammatory response in astrocytes is dependent on Toll-like receptor 4 (TLR4). In addition, Gu et al. [138] demonstrated that the expression of PD-related A53T α-synuclein mutation selectively in astrocytes disrupted astrocytic functions, including glutamate uptake and blood-brain barrier (BBB) regulation, as well as inducing microglial activation, which leads to inflammatory responses, and causes non-cell autonomous dopaminergic neurodegeneration in mice. Moreover, Yun et al. [139] reported that pathologic α-synuclein activated microglia to secrete IL-1α, TNF-α, and C1q, followed by the induction of reactive A1 astrocytes, which caused neurodegeneration. These data suggest that α-synuclein plays an indirect role in astrocyte-mediated neurodegeneration via microglial activation in PD. Taken together, these findings suggest that cell-to-cell α-synuclein transfer can activate astrocytes and microglia to produce inflammatory molecules, which is an important mechanism of neurodegeneration in PD (Figure 1).

The response of astrocytes to α-synuclein is not only a mechanism for the production of inflammatory cytokines, but also for the induction of mitochondrial dysfunction and oxidative stress. Chavarría et al. [140] examined the effects of different species of α-synuclein, including monomeric, oligomeric, and fibrillar forms of the protein, on cultured astrocytes. The incubation of astrocyte cultures with different α-synuclein species induced astrocyte activation; in particular, increased GFAP immunoreactivity was found in fibril- and oligomer-treated astrocytes. Astrocytes activated by α-synuclein treatment (monomeric, oligomeric and mainly fibrillar forms) were found to induce neuronal death. Similar to other studies, all types of α-synuclein were found to induce the expression of pro-inflammatory cytokines in astrocytes; conversely, only oligomers induced mitochondrial dysfunction in astrocytes and significantly increased the production of extracellular hydrogen peroxide by these cells [140]. Furthermore, Díaz et al. [141] reported that α-synuclein enhanced the opening of connexin 43 hemichannels and pannexin-1 channels in mouse cortical astrocytes, which was linked to the activation of cytokines, p38 MAP kinase, inducible nitric oxide (NO) synthase, cyclooxygenase (COX)-2, the intracellular free Ca^2+^ concentration, and purinergic and glutamatergic signaling. In fact, the α-synuclein-induced opening of hemichannels and pannexons resulted in alterations in Ca^2+^ dynamics, NO production, gliotransmitter release, mitochondrial morphology, and astrocyte survival. Recently, Sheng et al. [142] provided a new perspective on the pathogenesis of PD; erythrocyte-derived α-synuclein in extracellular vesicles was found to readily cross the BBB and accumulate in astrocyte end-feet, where they impaired glutamate uptake, likely via interaction between EAAT2 and oligomeric α-synuclein.

## 5. Astrocyte Dysfunction in Parkinsonism

The loss of normal astrocyte roles (loss of function) is implicated in PD pathogenesis. Astrocytes construct the BBB in cooperation with endothelial cells and pericytes. The BBB disruption is thought to contribute to PD progression, because increased BBB permeability is linked to the infiltration of systemic inflammatory mediators into the brain, which can promote microglial activation and dopaminergic neurodegeneration. Gray et al. [143] reported an increase in the permeability of the BBB in the putamen of patients with PD. Astrocyte end-feet cover over 99% of cerebral capillaries and express various receptors, transporters and channels. Astrocytes are known to play a pivotal role in BBB maintenance by modifying the formation of tight junction in endothelial cells via the release of regulatory factors, such as TGF-α and GDNF, and regulate brain microvascular permeability via astrocyte-endothelial communication [144]. Thus, astrocytes act as “gate keepers” to prevent peripheral immune cell infiltration into the brain. Accordingly, impairment in astrocyte-endothelial communication could be involved in the increased BBB permeability observed in PD patients. In addition, the regulation of water permeability by astrocytic water channels, aquaporin-4 (AQP4), in the BBB plays an important role in several physiological conditions in the CNS. Recently, the expression and regulation of AQP4 have been studied in several pathological situations, suggesting that AQP4 participates in the onset and progression of PD [145]. Xue et al. [146] reported that AQP4-deficient mice displayed significantly stronger microglial inflammatory responses and a markedly greater loss of dopaminergic neurons after MPTP injection. The mechanism is that TGF-β1, a key suppressive cytokine in PD onset and development, was decreased in AQP4 deficient mice after MPTP treatment, which resulted from the impairment of its generation by astrocytes. In addition, Sun et al. [147] demonstrated that AQP4 modulates astrocyte-to-microglia communication in neuroinflammation; AQP4 knockout mice exhibited gliosis (astrocytosis and microgliosis) in PD models produced by chronic MPTP injections, accompanied by an increase in the production of IL-1β and TNF-α in the midbrain.

EAAT2 dysfunction leads to extracellular glutamate accumulation and the onset of excitotoxicity due to excessive stimulation of excitatory amino acid receptors, which is associated with many neurodegenerative diseases. A recent study demonstrated that astrocytic GLT-1 deficiency in the SNpc induced parkinsonian phenotypes, progressive motor deficits and nigral DA neuronal death in mice [148]; GLT-1 knockdown induced the activation of astrocytes and microglia in the SNpc. These findings suggest that GLT-1 dysfunction contributes to PD pathogenesis. Glutamine plays an important role in brain energy metabolism and as a precursor for the synthesis of neurotransmitter glutamate and γ-aminobutyric acid. In particular, glutamine transport between astrocytes and neurons is a key factor in the glutamate-glutamine cycle. Manganese (Mn) is known to lead to neurological disorders characterized by early psychotic symptoms, frequently followed by chronic symptoms common to PD [149]. Previous studies found that Mn exposure disrupted astrocytic glutamine transporter expression and function [150,151]. Thus, the Mn-induced reduction of glutamine transport in astrocytes disrupts glutamate homeostasis and diminishes its availability to neurons, which may lead to impairment in glutamatergic neurotransmission and neurotoxicity.

As mentioned above, the antioxidative properties of astrocytes are extremely important for neuronal survival, especially for DA neurons. The Nrf2 signaling pathway followed by the induction of antioxidants, such as GSH and MTs, is essential in antioxidative defense. Therefore, the dysfunction of the Nrf2 system in astrocytes easily leads to dopaminergic neurodegeneration (Figure 1). Innamorato et al. [152] found that Nrf2 knockout mice showed exacerbated gliosis and dopaminergic neurodegeneration after injection with MPTP. L’Episcopo et al. [153] also found that aging downregulated Nrf2 expression and its activation in response to MPTP exposure. Furthermore, Lastres-Becker et al. [154] reported that human α-synuclein-expressing Nrf2 knockout mice exhibited exacerbated α-synuclein aggregation and the degeneration of nigral dopaminergic neurons, which is correlated with neuroinflammation and gliosis. Similarly, Nrf2 deficiency induced the microglial activation and production of inflammatory cytokines. As described above, the disruption of Nrf2 signaling in astrocytes leads to a reduction in GSH synthesis, which is linked to GSH depletion in neighboring neurons. Chinta et al. [155] reported that the depletion of GSH within dopaminergic neurons promoted a reduction in mitochondrial complex I activity and age-related dopaminergic neurodegeneration. These findings suggest that the reduction of the GSH-supplying ability of astrocytes could result in the selective inhibition of mitochondrial complex I and dopaminergic neuronal damage. MTs are strong antioxidative molecules produced by astrocytes. MTs also detoxify metal toxicity and regulate metal homeostasis. It has been reported that MTs are overexpressed in PD brains, in which iron accumulates [64]. Furthermore, recent studies have demonstrated that MTs may influence α-synuclein aggregation in PD [156]. Cu accumulates in the brain with aging and binds to α-synuclein, which initiates α-synuclein aggregation [157,158]. MTs bind Cu with a high affinity, suggesting that MTs play a role in Cu homeostasis [159]. Several studies have reported that MTs prevented the Cu-induced aggregation of α-synuclein [160,161]. Taken together, the findings suggest that the dysregulation of MTs may disturb metal homeostasis, leading to neurotoxicity. Indeed, we previously reported aggravated neurodegeneration in the nigrostriatal pathway in MT-1/-2 knockout PD model mice [162,163].

Various studies have demonstrated that the knockout or mutation of genes related to early-onset AR-PD promotes astrocyte dysfunction (loss of function) resulting in dopaminergic neurodegeneration. DJ-1 is a redox-sensitive protein with multiple functions, including mitochondrial physiology, protein transcription, proteasome regulation, and chaperone activity [164]. DJ-1 gene deletions and point mutations have been identified as one of the causes of AR-PD (PARK7) [77]. As mentioned above, DJ-1 is mainly expressed in astrocytes in the human brain, and acts as a sensor of oxidative stress [79,80]. Reactive astrocytes induce DJ-1 expression in response to oxidative stress and release the protein extracellularly to protect neurons [81,165]. Several studies have demonstrated that DJ-1 deficiency or mutation in astrocytes impairs astrocyte-mediated neuroprotection in parkinsonian models [166,167,168]. In addition, DJ-1 deficiency impairs glutamate uptake into astrocytes by altering EAAT2 expression [169], and reduces mitochondrial motility in astrocytes [166]. Moreover, DJ-1 selectively influences TLR4 to regulate astrocyte inflammation; DJ-1 deficient astrocytes induce proinflammatory mediators, such as COX-2 and IL-6 [170]. It has also been reported that IL-1β decreases the levels of DJ-1 and parkin in astrocytes [171].

Parkin (PARK2), an E3 ubiquitin ligase, forms a complex with PINK1 and DJ-1, and promotes the degradation of unfolded or misfolded proteins [172]. In addition, parkin and PINK1 have been identified as essential proteins for the removal of damaged mitochondria via autophagy (mitophagy) [96,97,98]. Therefore, parkin deficiency induces aberrant ubiquitination and compromised mitochondrial integrity, leading to neuronal dysfunction and degeneration. Constitutive parkin expression is higher in neurons than in astrocytes. However, astrocytes increase parkin expression during unfolded protein stress [173]. Solano et al. [174] examined the effects of parkin protein loss on the response of astrocytes to oxidative stress in parkin-knockout mice. As a result, parkin deficiency was found to impair GSH synthesis in astrocytes and showed the neurodegenerative pathogenesis of parkin-linked AR-PD. These results suggest that parkin deficiency results in the abnormal function of astrocytes, making dopaminergic neurons vulnerable to oxidative stress. In addition, it has also been reported that parkin-knockout astrocytes exhibit increased ER stress, cytokine production, and decreased astrocytic secretion of neurotrophic factors [175]. Moreover, severe mitochondrial damage was observed in mesencephalic astrocytes from parkin-knockout mice [176].

PINK1 is a serine/threonine kinase, whose mutation is associated with AR-PD (PARK6) [177]. PINK1 contributes to astrocyte development and proliferation, facilitating the autophagic degradation of damaged mitochondria [178,179]. PINK1 also regulates astrocytic inflammatory response, where the loss of PINK1 increases iNOS, NO, TNF-α, and IL-1β in astrocytes under neuroinflammatory conditions [180]. Moreover, it has been reported that PINK1-knockout astrocytes exhibit proliferation defects, which appeared to be linked to mitochondrial defects, increased intracellular ROS levels, decreased glucose-uptake capacity, and decreased ATP production [179]. These observations suggest that PINK1 deficiency promotes astrocytic dysfunction. In addition, parkin and PINK1 cooperate to degrade impaired mitochondria. Therefore, the dysfunction of PINK1-parkin-mediated mitophagy in astrocytes can lead to the disruption of mitochondrial maintenance.

Mutation within LRRK2 is a common cause of autosomal-dominant PD (PARK8). Although the physiological functions of LRRK2 remain unclear, it has been reported that the protein localizes to vesicular structures such as endosomes and lysosomes, suggesting that LRRK2 functions as a regulator of the endolysosomal system [181]. Correlations between mutant LRRK2 and several pathogenic mechanisms linked to PD progression have been reported, with mutations within LRRK2 altering autophagy and, consequently, the accumulation of α-synuclein [182,183,184]. LRRK2 is constitutively expressed in not only neurons but also glial cells, astrocytes and microglia, in the human brain [185,186]. Astrocytic LRRK2 is also involved in the autophagy-lysosome pathway as well as neurons. Henry et al. [187] demonstrated that LRRK2 regulates lysosome size, number and function in astrocytes. The expression of LRRK2 G2019S, the most common pathological mutation, produced enlarged lysosomes and diminished the lysosomal capacity of astrocytes. As mentioned above, a recent study demonstrated that iPSC-derived astrocytes from familial mutant LRRK2 G2019S PD patients exhibited dysfunctional chaperone-mediated autophagy, impaired macroautophagy, and progressive α-synuclein accumulation, which caused dopaminergic neurodegeneration [113]. Taken together, these findings indicate that the dysfunction of the autophagy-lysosome pathway in astrocytes may be implicated in PD pathogenesis.

ATP13A2 is a transmembrane lysosomal P5-type ATPase, whose mutation results in the Kufor-Rakeb Syndrome, a form of AR-PD (PARK9) [188]. Missense or truncation in the Atp13a2 gene impairs lysosomal function. Qiao et al. [189] reported that ATP13A2 deficiency induced astrocytic inflammation via NLRP3 inflammasome activation, exacerbating dopaminergic neuronal damage after MPTP treatment. In addition, a recent study demonstrated that the loss of ATP13A2 function in astrocytes could contribute to neuronal α-synuclein pathology by using iPSC-derived dopaminergic neurons and astrocytes from healthy subjects and patients carrying mutations in lysosomal ATP13A2 [190]. The uptake and degradation of α-synuclein were reduced in ATP13A2-mutated astrocytes, which resulted in increased α-synuclein transmission between DA neurons.

Gaucher disease (GD) is a lysosomal storage disorder caused by a mutation in the glucocerebrosidase 1 (GBA1) gene, resulting in the deficiency of the enzyme glucocerebrosidase (GCase). Recently, patients with PD or the related disorder dementia with Lewy bodies were found to be more likely to carry a mutation in GBA1 compared to controls [191]. Aflaki et al. [192] reported that iPSC-derived astrocytes from GD patients showed deficient GCase activity, levels, and substrate accumulation. In addition, astrocytes mutated within GBA1 manifested broad deficits in lysosomal function and immune dysfunction [193]. Furthermore, the GBA1-mutated astrocytes exhibited impaired cathepsin D activity, leading to α-synuclein accumulation, and inflammatory response. Thus, GBA1-mutated astrocytes appear to play a role in α-synuclein accumulation, contributing to neuroinflammation.

To date, neurotoxins, such as MPTP and 6-OHDA, have been used to produce PD models. In MPTP models, astrocytic activation occurs in parallel to dopaminergic cell death. Meanwhile, GFAP-expression remains upregulated even after most dopaminergic neurons have died [194], indicating that astrocytic reactions occur after neuronal cell death [195]. Astrocytes take up MPTP and convert it into neurotoxic 1-methyl-4-phenylpyridinium (MPP^+^), which inhibits the mitochondrial complex I [196]. Because MPP^+^ is taken up into neurons via the DAT [16], neurotoxins are able to attack dopaminergic neurons specifically. Since astrocytes also express DAT, it is suggested that MPP^+^ also affects astrocytes. Boyalla et al. [197] demonstrated that MPP^+^ decreased the ATP levels and increased ROS in astrocytes. These data suggest that MPP^+^ impairs energy production in astrocytes and increases oxidative stress, leading to neuronal damage. In addition, Chuang et al. [198] also reported that MPP^+^ induced astrocyte apoptosis and oxidative stress. Another neurotoxin, 6-OHDA, is also taken up by dopaminergic neurons via DAT, and then oxidized to produce radicals, thereby inhibiting mitochondrial complex I [199]. Therefore, 6-OHDA could also be captured by astrocytes, affecting their function. Indeed, Gupta et al. [200] demonstrated that 6-OHDA decreased mitochondrial dehydrogenase activity and mitochondrial membrane potential, and increased ROS levels, caspase-3 mRNA level, chromatin condensation, and DNA damage, which induced apoptotic cell death in astrocytes. Taken together, these findings suggest that neurotoxin-induced dopaminergic neurodegeneration could occur as a result of astrocyte dysfunction, which can cause oxidative stress, neuroinflammation and excitotoxicity.

## 6. Neuroprotective Effects of Astrocyte and Therapeutic Strategy for PD

### 6.1. Neuroprotective Response of Astrocytes against Oxidative Stress

As mentioned above, astrocytes possess multiple neuroprotective properties, in particular via the antioxidative machinery Nrf2/Keap1 signaling pathway. Our previous studies [13] demonstrated that excessive exposure to DA, which induces oxidative stress, promoted the nuclear translocation of Nrf2 and upregulated the expression of MT-1/-2 in striatal astrocytes, which are mediated by DA uptake via DAT. These astrocytic responses against oxidative stress protected DA neurons in parkinsonian models. Notably, the Nrf2 signaling pathway in astrocytes responded promptly to oxidative stress. Thus, nuclear Nrf2 could bind to ARE of MT-1 gene and promote MT-1 expression 1 h after DA exposure.

The cystine/glutamate exchange transporter xCT, which is mainly expressed in astrocytes, plays a vital role in GSH synthesis. Cystine is taken up via xCT into astrocytes, while, simultaneously, glutamate is exported into the extracellular space. The released glutamate is then taken up by astrocytes via GLT-1. Thus, GLT-1 and xCT cooperate for continuous cystine uptake, followed by GSH synthesis, to protect neurons against oxidative stress [201]. Excess extracellular glutamate blocks xCT, leading to GSH depletion. The loss of GSH causes ROS accumulation, and eventually cell death. In our previous studies, xCT expression was upregulated in reactive astrocytes in the striatum of parkinsonian mice [202]. Consistently, Bentea et al. [203] reported that xCT expression was increased in the striatum but decreased in the SN of MPTP-injected PD mice. In addition, Massie et al. [204] reported that xCT expression was increased in the lesioned striatum of PD model rats 3 weeks after 6-OHDA injection, and returned to normal at 12-weeks post-lesion. Moreover, the same research group also demonstrated that striatal GLT-1 expression and its activity in PD rats were upregulated at 3 and 12-weeks post-lesion, whereas no changes were observed at 5 weeks [205]. These findings suggest that astrocytes upregulate xCT and GLT-1 expression in response to oxidative stress.

### 6.2. Upregulation of Neuroprotective Properties in Astrocytes

**Figure 2:**
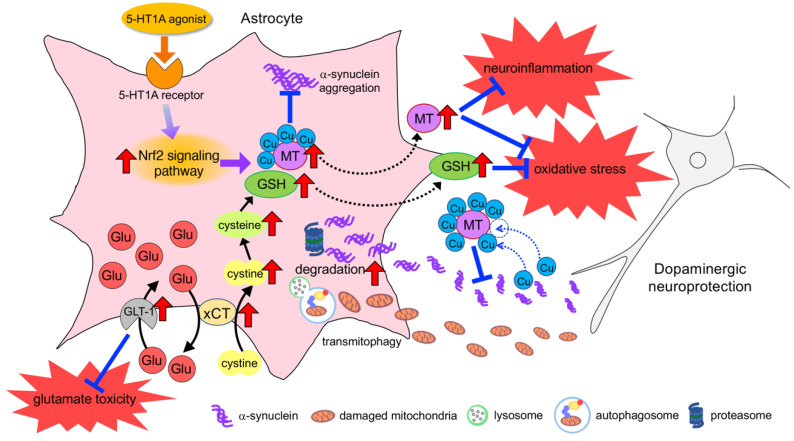
Targeting molecules in astrocytes for dopaminergic neuroprotection. Upregulation of astrocytic xCT increases GSH synthesis leading to neuroprotection against oxidative stress. GLT-1 plays an important role in inhibiting glutamate toxicity. GLT-1 and xCT cooperate for continuous cystine uptake followed by GSH synthesis. MTs bind Cu with a high affinity and inhibit Cu-induced α-synuclein aggregation. The stimulation of 5-HT1A receptor on astrocytes activates the Nrf2 signaling pathway followed by the upregulation of antioxidants including GSH-related molecules and MTs. Astrocytes engulf the aggregated α-synuclein and damaged mitochondria released from neuronal cells and degrade these neuronal waste via the proteasome and autophagy pathways.

To date, various studies have demonstrated neuroprotective approaches by targeting astrocyte function in parkinsonian models [9,206]. There is a consensus that the activation of the Nrf2 signaling pathway in astrocytes provides neuroprotection by producing various antioxidative molecules (Figure 2). Chen et al. demonstrated that the astrocyte-specific overexpression of Nrf2 almost completely attenuates MPTP neurotoxicity [74]. In addition, Gan et al. [207] reported that the overexpression of Nrf2 in astrocytes delayed the appearance of motor dysfunction and α-synuclein aggregation in α-synuclein mutant (A53T) transgenic mice. Various studies have revealed that phytochemicals, such as curcumin, sulforaphane, and resveratrol, could activate Nrf2 and exert neuroprotective effects against oxidative stress in animal models of various neurological disorders [208,209,210]. Our previous studies demonstrated that serotonin 1A (5-HT1A) agonist (R)-(+)-8-hydroxy-2-(di-*n*-propylamino) tetralin hydrobromide (8-OH-DPAT) promoted astrocyte proliferation via the secretion of S100β and the upregulation of the antioxidative molecule MT-1/-2 via Nrf2 nuclear translocation. The 5-HT1A agonist exerted dopaminergic neuroprotection in parkinsonian models [75]. In addition, our recent experiments showed that the antiparkinsonian drug rotigotine, which is a DA agonist and a 5-HT1A partial agonist, induced astrocyte proliferation and upregulated MT-1/-2 by activating the Nrf2 signaling pathway in astrocytes, and ameliorated dopaminergic neurodegeneration in parkinsonian mice [211]. We confirmed the upregulation of astrocytic antioxidative properties and neuroprotective effects of rotigotine via 5-HT1A receptors. In addition, 5-HT1A agonist-induced astrocyte proliferation also leads to the upregulation of antioxidative properties; indeed, we observed an increase in the levels of GSH after 8-OH-DPAT treatment. Thus, the development of pharmacological agents that target 5-HT1A receptors on astrocytes could provide a promising therapeutic strategy for neuroprotection in the treatment of PD (Figure 2).

As mentioned above, GSH can be regarded as an important neuroprotectant especially in dopaminergic neurons [58]. However, reaching an effective concentration of GSH in the brain by GSH administration is challenging due to the instability of small peptides and the degradability of amino acids. Furthermore, a recent clinical study showed that weekly intravenous infusions of N-acetyl-cysteine (NAC), the prodrug to L-cysteine, a GSH precursor (50 mg/kg), in combination with oral doses (500 mg twice per day) for 3 months significantly increased DAT binding (DaTscan) in the caudate and putamen of patients with PD, along with significantly improved PD symptoms [212]. These results suggest that NAC may positively affect the dopaminergic system in PD patients, with the corresponding positive clinical effects. Thus, astrocytic xCT upregulation could be a promising approach. Shih et al. [49] demonstrated that the overexpression of xCT in astrocytes increased GSH synthesis and supply, as well as protecting neurons against oxidative stress. In previous studies, we demonstrated that antiepileptic drugs, namely zonisamide and levetiracetam, increased xCT expression and GSH synthesis in striatal astrocytes, thereby preventing dopaminergic neurodegeneration in parkinsonian mice [202,213]. In addition, zonisamide has been reported to increase the number of S100β-positive astrocytes [214], as well as upregulate the mRNAs encoding astrocytic antioxidative and neurotrophic factors: vascular endothelial growth factor, proliferating cell nuclear antigen, MT-2, and SOD [215]. Furthermore, DA agonists, such as cabergoline and ropinirole, increase the expression of GSH-related enzymes and the GSH content, ameliorating dopaminergic neuronal loss in parkinsonian models [216,217]. A recent study demonstrated that the astrocytic DA D2 receptor regulates GSH synthesis via pyruvate kinase M2 (PKM2)-mediated Nrf2 transactivation [218]. Thus, the DA D2 agonist may activate PKM2-Nrf2 signaling in astrocytes. In addition, we found that L-theanine, a major free amino acid component of green tea, also exerts protective effects on DA neurons against oxidative stress by upregulating GSH expression in astrocytes [219].

Various studies have reported that GDNF exerts neuroprotective effects in parkinsonian models. However, the molecular size of GDNF prevents it from penetrating the BBB. The DA agonists pramipexole and ropinirole have been reported to upregulate GDNF and BDNF secretion from mesencephalic astrocytes, thereby ameliorating dopaminergic neurodegeneration after MPP^+^ treatment [220]. In addition, Ohta et al. [221] examined the stimulatory effects of various DA agonists, such as bromocriptine, cabergoline, pergolide, and SKF-38393, on the synthesis and secretion of NGF, BDNF, and GDNF in cultured astrocytes. As a result, these DA agonists were found to increase the levels of NGF and GDNF in the medium of cultured astrocytes. Pramipexole exerts neuroprotective effects against ubiquitin-proteasome impairment by upregulating BDNF from astrocytes [222]. Moreover, it has been reported that the overexpression of CDNF in astrocytes ameliorates cell damage and inflammatory cytokine secretion induced by ER stress [223]. These results suggest that the upregulation of neurotrophic factor secretion from astrocytes shows potential as a target of neuroprotection.

The modulation of DJ-1 expression in astrocytes could be a suitable target for neuroprotection. Indeed, it has been reported that astrocyte-specific DJ-1 overexpression protects dopaminergic neurons in rotenone-induced PD model rats [224]. In animals overexpressing DJ-1 in astrocytes, nigrostriatal DA neurodegeneration, neuronal oxidative stress and microglial activation were dramatically reduced. In addition, α-synuclein accumulation and phosphorylation were decreased in DA neurons, while the expression of LAMP-2A, a marker of chaperone-mediated autophagy, was increased. These data indicate that astrocyte-specific DJ-1 overexpression protects neurons against the pathological features of PD and provides evidence of a non-cell-autonomous neuroprotective function of astrocytic DJ-1.

Drugs that upregulate astrocytic GLT-1 may be prospective neuroprotectants by preventing excitotoxicity. Ceftriaxone, a β-lactam antibiotic, was discovered to be capable of increasing GLT-1 expression and exerting neuroprotective effects [225]. Leung et al. [226] demonstrated that ceftriaxone administration ameliorated motor deficits, determined by measuring the grip strength and the number of apomorphine-induced contralateral rotations, as well as protecting dopaminergic neurons in 6-OHDA-lesioned PD model rats. In addition, ceftriaxone inhibited the reduction of GLT-1 expression in the lesioned striatum of PD rats. Furthermore, ceftriaxone was found to improve cognition and prevent neurodegeneration in the hippocampus in a MPTP-induced animal model of PD dementia [227]. Riluzole, a Na^+^ channel blocker with anti-glutamatergic activity, is used to treat amyotrophic lateral sclerosis. Previous studies demonstrated that riluzole elevated the levels of GLT-1 enhanced its activity [228], as well as stimulating the synthesis of nerve growth factor, BDNF, and GDNF [229] in cultured astrocytes. In addition, the administration of riluzole antagonized the decrease in DA and metabolite levels, as well as preventing dopaminergic neurodegeneration in MPTP-injected mice [230].

Copper is needed for a multitude of processes including the synthesis of neurotransmitters, the transformation of energy within mitochondria, antioxidant defenses, and cell signaling [231,232]. Copper homeostasis is disrupted in the PD brain, and the levels of free Cu level are increased in the cerebrospinal fluid [233]. Neuromelanin-bound Cu is decreased in the SN of PD patients, suggesting that free Cu is increased, which leads to an acceleration of α-synuclein aggregation [157]. Since MTs can bind Cu with a high affinity, MTs are a potential target for attenuating Cu-induced α-synuclein aggregation (Figure 2). As mentioned above, we demonstrated that 5-HT1A agonists upregulated MT-1/-2 expression in astrocytes and promoted secretion into extracellular space [75,206,211]. Accordingly, 5-HT1A agonists can exert neuroprotective effects against not only oxidative stress but also α-synuclein toxicity.

As mentioned above, astrocytes engulf the aggregated α-synuclein and damaged mitochondria released from neuronal cells and degrade neuronal waste via the proteasome and autophagy (mitophagy) pathways [94,95,234]. Thus, phagocytic activity of astrocytes is important for neuroprotection (Figure 2). Shu et al. [235] recently reported that fluoxetine, a selective serotonin reuptake inhibitor (SSRI), promoted the autophagic flux unblocked by enhancing the fusion of autophagosomes with lysosomes in cultured astrocytes. In addition, this drug promoted mitophagy by increasing the colocalization of autophagosomes and mitochondria, eliminating damaged mitochondria. Moreover, fluoxetine has been reported to ameliorate behavioral and neuropathological deficits in a transgenic model mouse of α-synucleinopathy [236]. Konno et al. [237] reported that an antidepressant sertraline inhibited dynamin GTPases and decreased α-synuclein uptake by neuronal and oligodendroglial cells, suggesting therapeutic strategies aimed at reducing the propagation of protein misfolding. Sharma et al. [238] demonstrated that treatment with sertraline significantly attenuated motor dysfunction, oxidative stress, neuroinflammation, mitochondrial dysfunction and decreased striatal catecholamine levels in rotenone-induced parkinsonian rats. These results suggest that antidepressants could act as neuroprotectants in PD treatment, although cases of SSRI-induced parkinsonism have been documented [239].

## 7. Conclusions

Impaired astrocytes contribute to various pathogenic factors in PD, such as oxidative stress, neuroinflammation, α-synuclein toxicity, and mitochondrial impairment. Therefore, the normalization of astrocytic dysfunction or the upregulation of neuroprotective ability represents a therapeutic approach to prevent progressive neurodegeneration in PD. However, neurodegeneration accompanied by α-synuclein accumulation is observed in the central and peripheral nervous system in PD patients, and the mechanism underlying selective neuronal death in specific brain areas remains unknown. Astrocytes were recently recognized as morphologically and functionally diverse cells, with a reactivity that varies according to the brain region [240]. In addition, the diverse roles of astrocytes include modulation of neuronal survival via neuron-astrocyte interactions. In recent studies, we identified regional differences in the response of astrocytes and the induction of antioxidative molecules in astrocytes against oxidative stress, and found that region-specific features of astrocytes lead to region-specific vulnerability of neurons [241]. cDNA microarray analysis showed that 6-OHDA treatment upregulated the expression of Nrf2 and Nrf2-regulating molecules related to GSH synthesis in striatal astrocytes but not mesencephalic astrocytes. In addition, midbrain neurons co-cultured with striatal astrocytes were found to be more resistant to 6-OHDA than those with mesencephalic astrocytes. Furthermore, astrocyte conditioned media from 6-OHDA-treated striatal astrocytes showed a greater protective effect on 6-OHDA-induced neurotoxicity and oxidative stress than that from mesencephalic astrocytes. These results suggest that the region-specific response of astrocytes could determine neuronal vulnerability. Furthermore, with regards to astrocyte-microglia interactions, such as activated microglia-induced A1 astrocyte in neuroinflammation, regional differences in astrocyte-microglia interactions and their response in neurological conditions may contribute to the region-specific neuronal death observed in PD pathology.

PD is a complex, multi-system, neurodegenerative disorder, with PD patients exhibiting not only motor symptoms due to the loss of nigrostriatal dopaminergic neurons but also non-motor symptoms that precede motor symptoms [1]. Gastrointestinal dysfunction is a particularly prominent non-motor symptom of PD. Constipation appears approximately 10 to 20 years prior to the presentation of motor symptoms [242]. Pathologically, α-synuclein deposition is also observed in the peripheral nervous system of sporadic PD patients, including the intestinal myenteric plexus, gastric mucosa, cardiac sympathetic nerve, and skin nerve [243]. It is currently hypothesized that PD pathology propagates from the ENS to the CNS via the vagal nerve [4]. The ENS controls the gastrointestinal tract on the local level, in which astrocyte-like GFAP-positive stellate-shaped enteric glial cells (EGC) are found. EGC express various receptors for neurotransmitters and neuromodulators that allow them to respond to transmitters released from neurons [244]. Thus, the intimate physical association between enteric glia and neurons is highly reminiscent of the relationship between astrocytes and neurons in the CNS. EGC dysfunction in pathological conditions has been attributed to abnormal intestinal motor activity, which in turns causes constipation. In a recent study, we observed a reduction of GFAP-positive enteric glia in the intestine of a novel parkinsonian model produced by chronic systemic exposure to a low dose of rotenone (2.5 mg/kg/day) for 4 weeks [245]. The PD model also exhibited neurodegeneration of the intestinal myenteric plexus, accompanied by α-synuclein accumulation and gastrointestinal dysfunction [246]. In addition, the expression of MT-1/-2 in GFAP-positive enteric glia cultured cells from the intestine was found to decrease after exposure to rotenone. Furthermore, treatment with the coffee components caffeic acid and chlorogenic acid was found to inhibit the rotenone-induced reduction of MT/1-/2 expression in enteric glia and neurodegeneration in the myenteric plexus [245]. These results suggest that the upregulation of antioxidative molecules in the EGC prevents neurodegeneration in the ENS. Taken together, these findings suggest that astrocytes (astrocyte-like glial cells) contribute to neuronal function and survival not only in the CNS but also ENS. As a result, this review provides insights for the design of new therapies aimed at providing neuroprotection against neurological disorders.
